# Neurotrophic keratitis in autoimmune polyglandular syndrome type 1: a case report

**DOI:** 10.1186/s12886-020-01770-w

**Published:** 2021-01-07

**Authors:** Po-Ying Wu, Huai-Wen Chang, Wei-Li Chen

**Affiliations:** 1 Department of Ophthalmology, National Taiwan University Hospital, No. 7, Chung-Shan South Road, Taipei, Taiwan; 2 Advanced Ocular Surface and Corneal Nerve Regeneration Center, National Taiwan University Hospital, Taipei, Taiwan; 3 Department of Ophthalmology, College of Medicine, National Taiwan University, Taipei, Taiwan

**Keywords:** Autoimmune polyglandular syndrome type 1 (APS-1), Case report, In vivo confocal microscopy, Neurotrophic keratitis

## Abstract

**Background:**

Autoimmune polyglandular syndrome type 1 (APS-1) is a rare autosomal recessive disease. In patients with APS-1, the most frequently reported ocular manifestations are keratoconjunctivitis with dry eye and retinal degeneration. However, to our knowledge, no research studies have reported the relationship between APS-1 and neurotrophic keratitis (NK). Possible explanations such as limbus cell deficiency being the primary cause of APS-1 keratopathy are not applicable to our unusual case of the patient with APS-1 presenting as ocular surface disease with NK. Our case findings suggest a new explanation for the observed corneal pathology and a potential treatment for these patients.

**Case presentation:**

A 27-year-old woman was referred to our hospital because of intermittent blurred vision and recalcitrant ocular surface problems in both eyes for many years. She has a history of autoimmune polyglandular syndrome type 1 (APS-1), which includes hypothyroidism, hypoparathyroidism, hypoadrenalism, and hypogonadotropic hypogonadism. In vivo confocal microscopy clearly demonstrated significant degeneration of the sub-basal nerve plexus and stromal nerve bundles in her corneas bilaterally. She was diagnosed with severe NK and ocular surface disease caused by dry eye. Treatment included the application of therapeutic soft contact lenses and punctual occlusion; however, both treatments had a limited effect.

**Conclusion:**

Patients with APS-1 may have ocular surface disease and severe damage to corneal nerves. Regular follow-up and treatment focusing on the regeneration of corneal nerves is particularly important in these patients.

## Background

Autoimmune polyglandular syndrome (APS) is a rare autosomal recessive disease [1]. Due to immune intolerance, it causes several endocrine organ dysfunctions. APS can be categorized into three types: Type I (APS-1), Type II (APS-2), and X-linked immunodysregulation, polyendocrinopathy, and enteropathy (IPEX). APS-1 is characterized by the development of at least two of three cardinal components during childhood: chronic mucocutaneous candidiasis, hypoparathyroidism, and primary adrenal insufficiency (Addison disease). Alopecia, vitiligo, and dystrophies of the dental enamel and nails are also common. Ocular manifestations are often involved, which are mostly reported as keratoconjunctivitis with dry eye and retinal degeneration. Since the underlying mechanism leading to the corneal manifestations in these patients has not been well established, there is no standard treatment for APS-1–associated keratopathy [2]. We used in vivo confocal microscopy (IVCM) to confirm the significant abnormality of the sub-basal and stromal corneal nerves in a patient with APS-1. Such abnormalities on IVCM are not usually seen in patients with dry eye. The etiology of ocular pathology as a result of APS-1 has been elusive, although some have proposed a theory of limbal stem cell deficiency [3]. However, limbal stem cell deficiency does not completely explain the key finding in our report, which is neurotrophic keratitis (NK) in a patient with APS-1. To our best knowledge, the relationship between APS-1 and NK has not been documented in the literature. Our case proposes a new explanation for the corneal pathology and a potential treatment for these patients.

## Case presentation

**Fig. 1:**
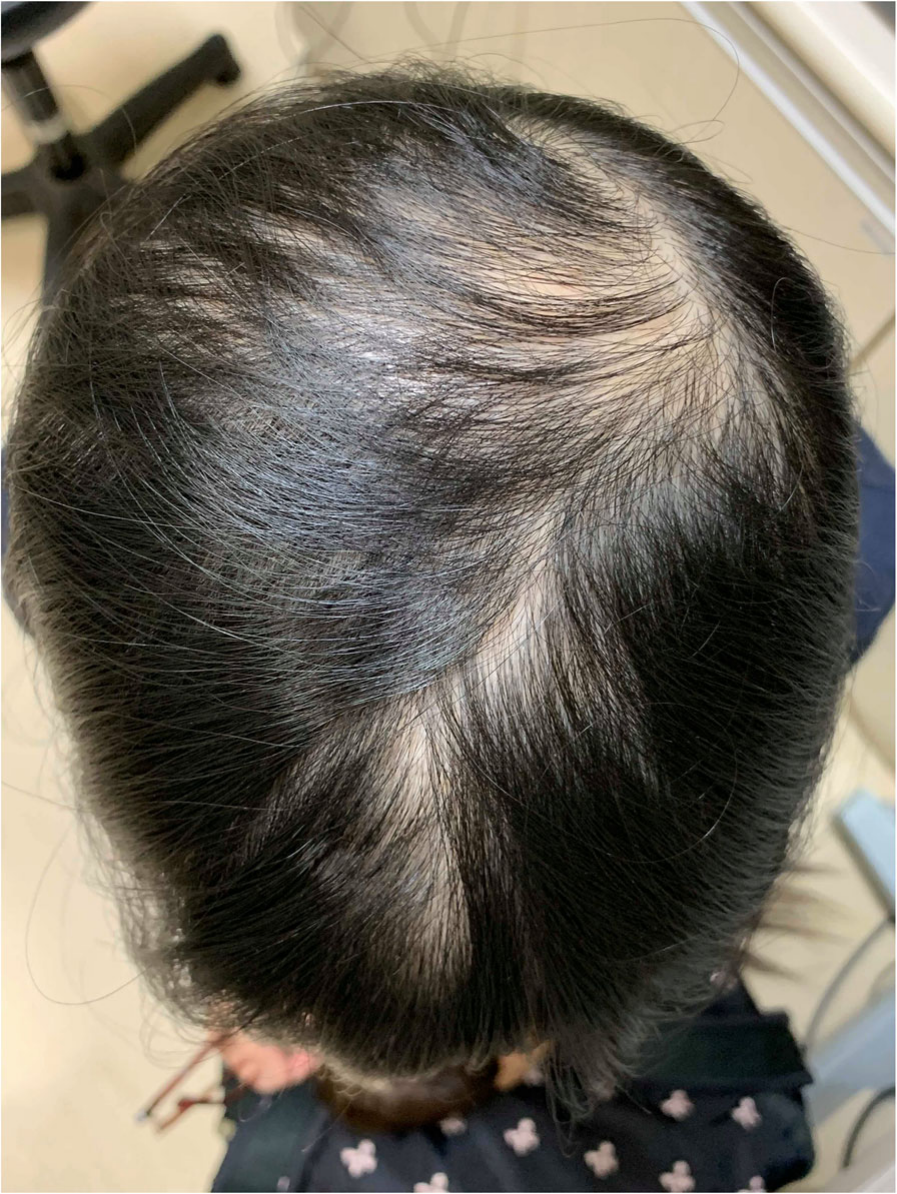
Alopecia was found

A 27-year-old woman was referred to our hospital because of chronic bilateral blurred vision and discomfort. She suffered from similar complaints 13 years ago. At that time, dry eye syndrome with bilateral corneal erosions was diagnosed. Her condition improved partially after topical treatment but was never completely cured. She had a past medical history of APS-1 including Addison disease, Hashimoto thyroiditis, hypoparathyroidism, and hypogonadotropic hypogonadism. She does not have diabetes mellitus. She has a short body height (147 cm) and low body weight (37 kg). Hair loss was also noted (Fig. 1). She wore soft contact lens for approximately 5 years and stopped wearing them for the last 3 years.

**Fig. 2:**
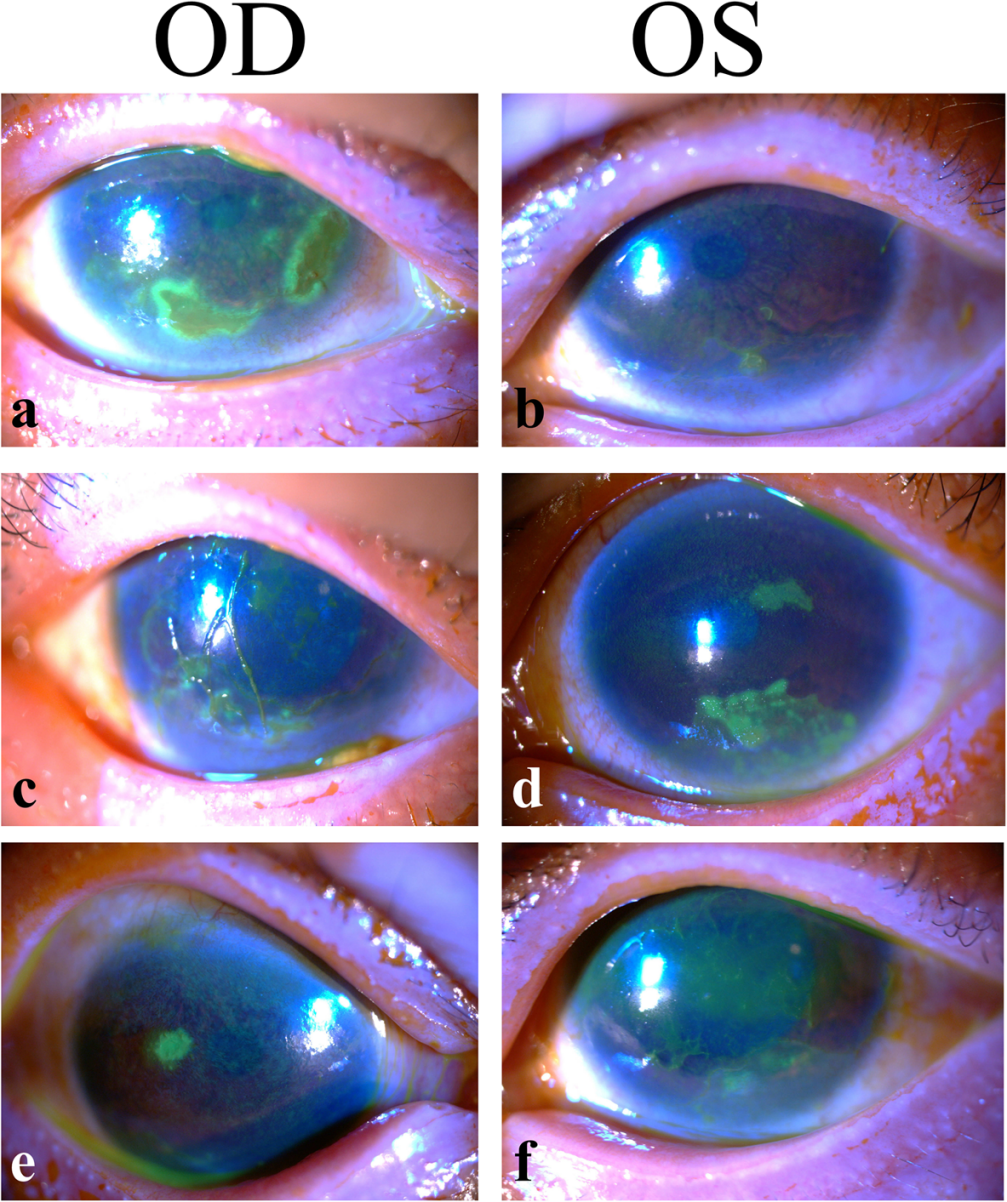
External eye photographs. On the first day visiting our clinic, slit-lamp examination with fluorescein staining revealed 2 patches of corneal erosions on the right eye (**a**), and diffuse superficial punctate corneal erosions with filaments on the left eye (**b**). Two months after treatment with lubricants, diffuse superficial punctate corneal erosions with filaments were found on the right eye (**c**), and 2 patches of corneal erosions were found in the left eye (**d**). Four months after treatment, there was 1 patch of corneal erosion in the right eye (**e**), and filamentary keratitis in the left eye (**f**)

On ocular examination, her corrected distance visual acuity was 20/40 in the right eye and 20/50 in the left eye. Intraocular pressure was normal. Slit-lamp examination revealed 2 patches of corneal erosions in the right eye (Fig. 2a). Diffuse superficial punctate erosions with filaments on the lower corneal surface were found in the left eye (Fig. 2b). Bilateral meibomian gland dysfunction was also noted. Schirmer testing under anesthesia revealed dry eye.

**Fig. 3:**
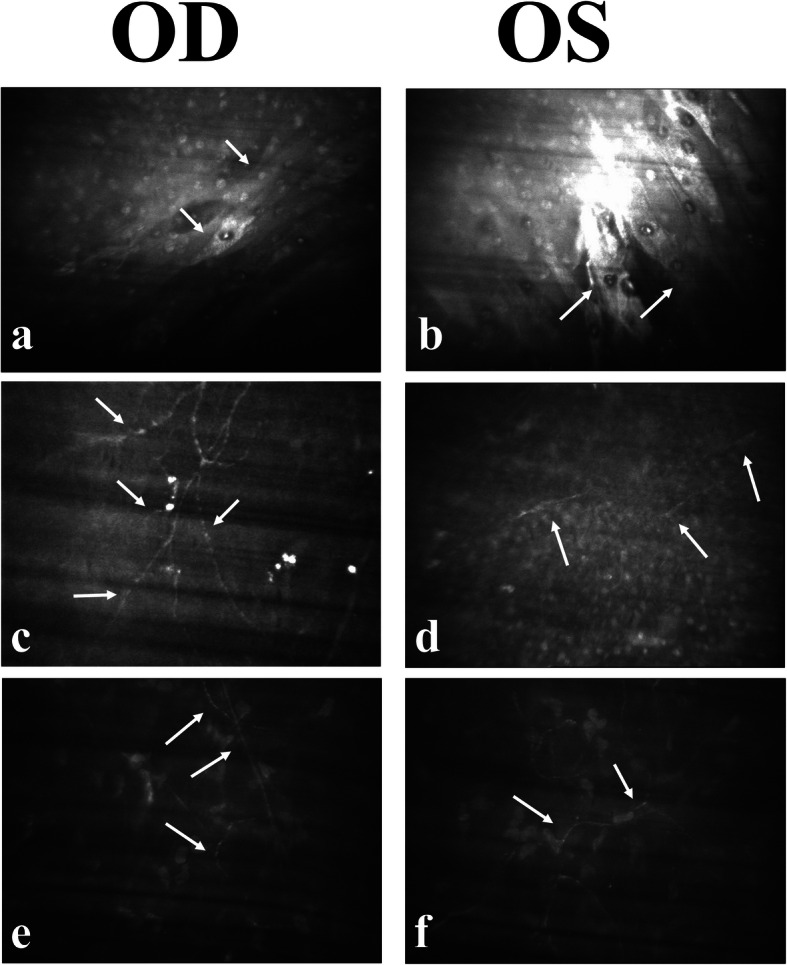
In vivo confocal microscopy findings of the cornea. **a**, **b** Squamous epithelial layer. White arrows indicate the elongated squamous epithelial cells with a tendency to slough off. **c**, **d** White arrows indicate the sub-basal nerve plexus, which was either tortuous and beading in the right eye (**c**), or short and fragmented in the left eye (**d**). The density of the nerve plexus was also severely affected. **e**, **f** White arrows indicate the corneal nerve bundle in the anterior stromal layer. Both eyes showed a significant decrease in the width, density, and length of the anterior corneal stromal nerve bundles compared with normal eyes

IVCM using Confoscan 3.4.1 (Nidek Technologies, Padova, Italy) was then performed and revealed significant abnormality of the sub-basal nerve plexus and stromal nerve bundles in both eyes. The squamous epithelial cells in the most superficial layer of the cornea were found to be elongated with a tendency to slough off easily, which highlights the poor differentiation of the corneal epithelial layer (Fig. 3a, b). The sub-basal nerve plexus was torturous and beading; it lost its parallel pattern in the right eye (Fig. 3c) and became short and segmented in the left eye (Fig. 3d). In both eyes, the stromal nerve bundles became very thin and torturous (Fig. 3e, f), which is quite different from the thick stromal nerve bundles in normal corneas. There was no significant change in the corneal endothelial layer. As a result, ocular surface disease caused by dry eye with severe NK was diagnosed.

In the following 4 months, the corneal surface problems persisted despite regular use of artificial tears, topical corticosteroids, cyclosporin, autologous serum eye drops, and nighttime ointment. Corneal erosions, superficial punctate erosions, and filamentary keratitis were found in both eyes (Fig. 2c-f). Treatment including therapeutic soft contact lenses, and punctual occlusion was later performed; both treatments had a limited effect. In the following 2 years, the corneal epithelial erosions relapsed intermittently.

## Discussion

APS-1 is a rare disease characterized by immune intolerance and caused by variations in the *AIRE* gene, which was first discovered in 1997 [4]. The *AIRE* gene encodes the autoimmune regulator protein, which is mainly expressed in thymic medullary epithelial cells and drives negative selection of self-recognizing T cells. Therefore, absence of the *AIRE* gene function may lead to spontaneous autoimmunity in either humans or mice [5].

Although the occurrence of keratopathy in APS-1 ranges from 25 to 50% [2, 6], there is no definite explanation for the underlying mechanism leading to the corneal pathology, and no detailed report on the change in the corneal nerves has been documented. The sites of *AIRE* gene expression in extrathymic locations are primarily within peripheral lymphoid tissues. Previous studies did not find direct *AIRE* gene expression in the eyes [7]. Nevertheless, a mouse model of *AIRE* gene deficiency showed that deficient mice experienced loss of nerve innervation in the cornea and the lacrimal gland, which was associated with spontaneous, CD4+ T-cell mediated exocrinopathy and aqueous-deficient dry eye [8]. This animal model finding is compatible with our findings in this patient with APS-1. Because APS-1 involves multiple endocrine disorders, it may cause ocular dysfunction as a result of multiple endocrine organ imbalances. For example, hypothyroidism can cause pathological changes on ocular and extraocular tissues, which include decreased tear secretion, periorbital edema, permanent loss of eyebrows and lashes, cataract formation, and rare corneal dystrophies [9]. Primary hypoadrenalism, also called Addison disease, may cause ptosis, blepharitis, blepharospasm, keratoconjunctivitis, corneal ulcers, episcleritis, cataract, and papilledema [9].

Our patient was diagnosed as having dry eye with NK because of the following constellation of findings: corneal manifestations, decreased corneal sensation, and morphological change of the corneal nerves, the latter of which was confirmed by IVCM. The most common causes of NK include herpesvirus infections, dry eye, and cranial neurosurgery. Other long-term ocular damage by contact lens misuse and topical medication or systemic conditions such as diabetic mellitus may also cause NK [10, 11]. To our knowledge, the link between NK and APS-1 has not been documented. The etiology of APS-1–associated keratopathy remains elusive [12]. Some researchers have proposed limbal cell deficiency as the primary cause of APS-1 keratopathy [3]. This theory presumes that the *AIRE* gene may play a role in limbal stem cell maintenance, though further investigation is needed to elucidate the exact relationship between the gene and limbal stem cells. Proponents of this theory advise keratolimbal allograft stem cell transplantation as a potential treatment for patients with APS-1 keratopathy. Results of these transplants have suggested promising outcomes in patients with APS-1 [13]. This theory could not be applied to our patient’s NK, as we did not find signs of limbal stem deficiency in our patient.

We hypothesized the possible cause of NK in our case as follows. First, the autoimmune imbalance in APS-1 may directly cause autoimmune peripheral neuropathy, including damage to corneal nerves. Corneal nerves are critical to maintaining ocular surface health. Loss of corneal innervation is commonly found in patients with Sjogren syndrome–associated dry eye [14]. Second, hypothyroidism in this patient can cause peripheral neuropathy, which could result in NK [15]. Third, the patient has secondary dry eye caused by APS-1 and has a history of contact lens wearing [10]. However, dry eye and short-term contact lens wearing typically cause sub-basal corneal nerve damage without impairing the stromal nerves. Our patient was found to have damage on both the sub-basal nerve plexus and the stromal nerves, which implied that NK may be the primary cause as a result of her endocrine dysfunction rather than being secondary to dry eye. *AIRE*-deficient mice models of corneal and lacrimal gland neuropathy that were found to exhibit autoimmune exocrinopathy strengthen our theory [8] that APS-1 may inherently cause neuropathy and therefore lead to APS-1 keratopathy secondarily.

There is another well-known endocrine problem that causes changes in corneal nerves. Multiple endocrine neoplasia (MEN) has been reported to cause prominent corneal nerves and is easily diagnosed from slit-lamp examination. However, in MEN, the clinical and pathological presentation of the corneal nerves is obviously different from our case, although both diseases involve the dysfunction of multiple endocrine systems. Future study should explore the reason for the different corneal presentations in these two diseases.

IVCM is a useful clinical tool for evaluating microscopic corneal structures [16]. It is especially useful to detect the degree of corneal nerve changes in patients. Changes in the pattern of corneal innervation evaluated by IVCM can be assessed using quantitative outcome measurement such as nerve length, density, and tortuosity [17]. Other less commonly used morphometric parameters include beading, branching, reflectivity, and fiber diameter. The morphology, length, and density of corneal nerves were found to be abnormal and decreased in our patient after repeated examination by IVCM. The impossibility of quantitative measurement of the corneal nerves implied severe corneal nerve damage.

To date, no standard treatment of APS-1–associated keratopathy has been established. Topical corticosteroids and nonsteroidal anti-inflammatory drugs have been reported to have satisfactory results in some but not all patients [6, 12]. APS-1–associated keratopathy can cause dry eye syndrome, and topical lubricants should be considered to relieve dry eye symptoms. Keratolimbal transplantation with systemic immunosuppression has been performed since stem cell deficiency is a possible underlying etiology of APS-1–associated keratopathy [3]. Additionally, keratoprostheses have been reported to be the therapy of choice in the late stages of APS-1–associated keratopathy [2, 12]. Overall, the prognosis of an APS-1–associated keratopathy can be severe and poor. In our patient, we found that damage of the corneal nerves may play an important role in the pathogenesis of APS-1–associated keratopathy, which has long been neglected. Neurotrophic factors, such as autologous serum, platelet-rich plasma, human platelet lysates, or nerve growth factor should be considered if conventional treatment has failed before directly initiating aggressive surgical treatment [17, 18].

In conclusion, our case demonstrates bilateral recalcitrant corneal surface problems with significant damage of the sub-basal and stromal corneal nerves in patients with APS-1. IVCM can provide helpful information to make the proper diagnosis. Accordingly, topical neurotrophic agents should be considered in dealing with recalcitrant corneal erosions in these patients if conventional treatment fails.

## Data Availability

The datasets used and/or analyzed during the current study are available from the corresponding author on reasonable request.

## Abbreviations

APS: Autoimmune polyglandular syndrome
IVCM: In vivo confocal microscopy
MEN: Multiple endocrine neoplasia
NK: Neurotrophic keratitis
